# Supplementary material for: Generation and Characterization of a CE1-Modified mCherry-Expressing Influenza A Virus for In Vivo Imaging and Antiviral Drug Evaluation
Source: Viruses. 2025 Nov 24;17(12):1537. doi: 10.3390/v17121537 (PMC12737787; doi:10.3390/v17121537)
Supplement: Supplementary file 1 [file viruses-17-01537-s001.zip › Supplementary Material.pdf]

Supplementary Material

Table S1 .Primers

Primer name	Primer sequence (5′-3′)
pPOLI-NS-F	GGCAGCGGCGCGACCAAC
pPOLI-NS-R	TCCGGACCCAACTTCGCT
mCherry-F	GAAGCGAAGTTGGGGTCCGGAATGGTGAGCAAGGGCGAGG
mCherry-R	AAGTTGGTCGCGCCGCTGCCCTTGTACAGCTCGTCCATGCC
M-F	CTTCTAACCGAGGTCGAAAC
M-R	CGTCTACGCTGCAGTCCTC

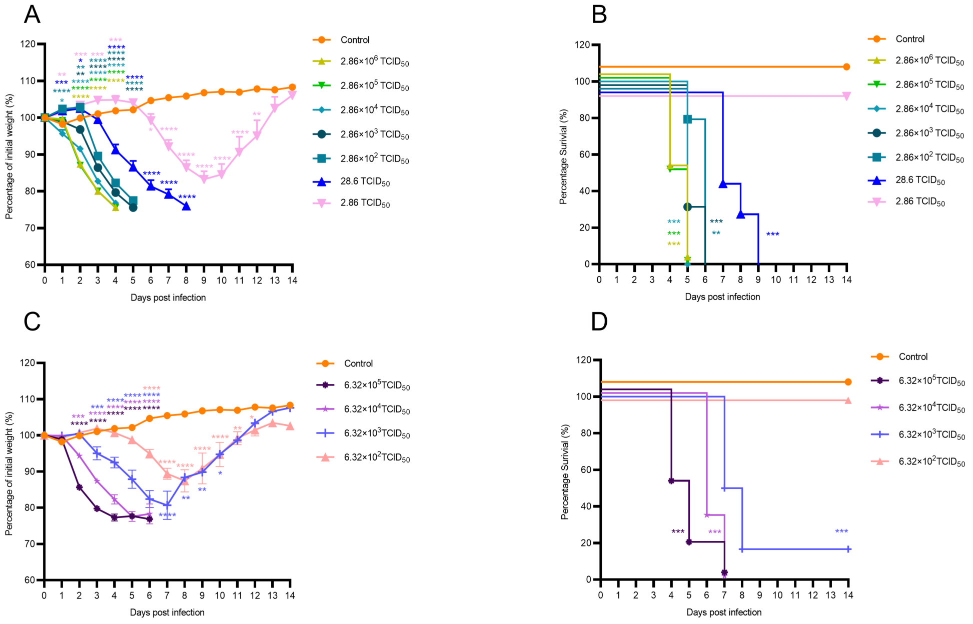

Figure S1. Pathogenicity of H1N1-PR8 and H1N1-PR8-NSCE1-mCherry in BALB/c mice. (**A**) Body weight changes of mice infected with H1N1-PR8. *n* = 6, data was presented as mean ± SEM. 2.86×10^6 TCID$_{50}$ vs. control; 2.86×10^5 TCID$_{50}$ vs. control; 2.86×10^4 TCID$_{50}$ vs. control; 2.86×10^3 TCID$_{50}$ vs. control; 2.86×10^2 TCID$_{50}$ vs. control; 28.6 TCID$_{50}$ vs. control; 2.86 TCID$_{50}$ vs. control: *$P < 0.05$, **$P < 0.01$, ***$P < 0.001$, ****$P < 0.0001$, one-way ANOVA. (**B**) Survival rates of mice infected with H1N1-PR8. *n* = 6, data was presented as mean ± SEM. 2.86×10^6 TCID$_{50}$ vs. control: ***$P < 0.001$; 2.86×10^5 TCID$_{50}$ vs. control: ***$P < 0.001$; 2.86×10^4 TCID$_{50}$ vs. control: ***$P < 0.001$; 2.86×10^3 TCID$_{50}$ vs. control: ***$P < 0.001$; 2.86×10^2 TCID$_{50}$ vs. control: **$P < 0.01$; 28.6 TCID$_{50}$ vs. control: ***$P < 0.001$, one-way ANOVA. (**C**) Bodyweight changes of mice infected with H1N1-PR8-NSCE1-mCherry. *n* = 6, data was presented as mean ± SEM. 6.32×10^5 TCID$_{50}$ vs. control; 6.32×10^4 TCID$_{50}$ vs. control; 6.32×10^3 TCID$_{50}$ vs. control, 6.32×10^2 TCID$_{50}$ vs. control: *$P < 0.05$, **$P < 0.01$, ***$P < 0.001$, ****$P < 0.0001$, one-way ANOVA. (**D**) Survival rates of mice infected with H1N1-PR8-NSCE1-mCherry. *n* = 6, data was presented as mean ± SEM. 6.32×10^5 TCID$_{50}$ vs. control: ***$P < 0.001$; 6.32×10^4 TCID$_{50}$ vs. control: ***$P < 0.001$; 6.32×10^3 TCID$_{50}$ vs. control: ***$P < 0.001$, one-way ANOVA.

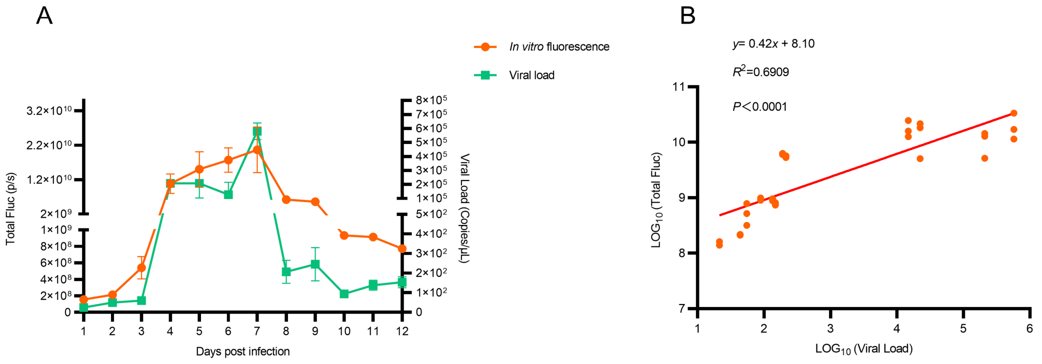

Figure S2. Correlation between in vitro fluorescence intensity and viral load. (**A**) Fluorescence signals and viral load in isolated lung tissues. n = 3, data was presented as mean ± SEM. (**B**) Correlation between in vitro fluorescence intensity and viral load.
